# Preferences for birth center care in the Netherlands: an exploration of ethnic differences

**DOI:** 10.1186/s12884-017-1254-3

**Published:** 2017-03-06

**Authors:** Dominique Lescure, Sanneke Schepman, Ronald Batenburg, Therese A. Wiegers, Ellen Verbakel

**Affiliations:** 1 NIVEL, Utrecht, The Netherlands; 2 Ministry of Health, The Hague, The Netherlands; 3 Radboud University, Nijmegen, The Netherlands

**Keywords:** Birth center, Patient preferences, Ethnicity, Socioeconomic factors

## Abstract

**Background:**

To examine the preferences for comprehensive services and facilities in a new proposed birth center which will be established in a large Dutch city, specifically among pregnant women from different ethnic backgrounds.

**Methods:**

The analyses of this study were based on a survey among 200 pregnant women living in The Hague, the Netherlands in 2011. Multiple linear regression was applied to analyze if preferences differ by ethnic background, controlling for various other predictors.

**Results:**

Pregnant women had relatively strong preferences for comprehensive services and facilities to be offered by the new proposed birth center compared to both other dimensions of birth center care: extensive practical information and comfortable accommodation. With regard to ethnic differences, non-Dutch women had higher preferences for comprehensive care compared to Dutch women. This difference between Dutch and non-Dutch women increased with their level of education.

**Conclusions:**

Especially for non-Dutch women, birth centers that are able to provide comprehensive services and facilities can potentially be a good setting in which to give birth compared to hospitals or at home. In particular, higher educated non-Dutch women had a preference for the personalized care that could be offered by this new birth center.

## Background

In the Netherlands and a number of other countries, birth centers are considered to be a relatively new type of facility and service through which birth care quality can be improved [1–4]. In particular, hospital-based centers, or alongside centers that are located in close proximity to hospitals, are designed to provide an intermediate option of care between home and hospital birth. In these centers the mutual collaboration and/or affiliation with a hospital (for births with medical complications) is facilitated. Moreover, these centers are expected to provide a safe and easily accessible place of birth as well as personalized care that relies on meeting specific health needs. The premise of birth center care is to optimize the involvement of women in planning their own pregnancy and birth care by providing flexible options [5–8].

Birth centers are emerging in countries such as Sweden, Germany and the United Kingdom and the demand for these healthcare facilities is increasing [9–11]. A Dutch study showed that women are positive about birth centers because of the safe and convenient feeling, nice atmosphere and reassurance that medical help is directly available [12]. Moreover, women who gave birth in a birth center felt in control, which is desired by many women and is known to be associated with higher satisfaction with the birth experience [13, 14]. The same Dutch study, however, also showed that some women disliked their experience in a birth center either because of busy birth attendants or because they were expected to leave quickly after the birth [12].

In the Netherlands, the first birth centers were established in 1883 in order to contribute to the education of midwives and to provide poor women with a safe place to give birth. Over the past century, the aim shifted towards a safe way to avoid ‘high-tech’ obstetrics in hospitals with low-risk pregnancies [8]. In addition, during the beginning of the twenty-first century, many birth centers were established in the Netherlands as an answer to the problem of a shortage of midwives and a resulting increase in hospital births^1^ [8]. Despite the emergence of birth centers, the Netherlands still has a high percentage of home births, which is one of the features that makes the Dutch system of maternity care unique in the world [15, 16]. To date, it remains unclear whether the expectations of female clients are better met in birth centers than in hospital or home births and if the offered birth care connects to the needs of different social groups, such as non-Dutch women, including first, second and third generation immigrants [6, 8, 17].

In 2009, the Steering Committee on Pregnancy and Birth^2^ [18] advised the Dutch government to focus particularly on pregnant women in disadvantaged situations, e.g., non-Dutch women. Non-Dutch women could experience problems with regard to the use of health care due to their different cultural backgrounds and unfamiliarity with the Dutch maternity care system [2, 19, 20]. This is accompanied by the problem that information about the necessity and possibilities of maternity care assistance^3^ does not reach them sufficiently [21, 22]. Consequently, these vulnerable groups underuse maternity care and, in addition, there is a mismatch between their specific care needs and the actual provided maternity care. This is problematic as non-Dutch women in the Netherlands face the highest risk of poor health outcomes for themselves and their (unborn) children [2]. Health (i.e., pregnancy) outcomes for this group can be improved by narrowing the information gap and offering customized care with specific attention to their medical, psycho-social and social problems [2].

Because of their adaptability to meet the needs of non-Dutch women in disadvantaged situations, birth center projects were mostly initiated in the urban areas of the Netherlands and cities such as The Hague. As the third largest city of the Netherlands, The Hague has a high proportion of Western (15.6%) and non-Western immigrants (34.4%). Many of them have a low socio-economic status (SES) and live in disadvantaged areas of the city [23]. Moreover, the perinatal mortality figures are highest here compared to other cities in the Netherlands [2].

The birth center project that was initiated in The Hague will be specifically focused on the collaboration between primary and secondary birth care^4^ and on better meeting the needs of pregnant women in disadvantaged situations. The provided care will not only be focused on birth care provision during pregnancy and childbirth, but also during the postpartum period. Moreover, the project has the intention to engage all healthcare professionals who are involved in care during pregnancy, childbirth and the postpartum period to be able to offer the care that is needed; this will result in the best possible care in every situation. This means that a broad range of healthcare professionals such as gynecologists, midwives, general practitioners and maternity care assistants will actually be present in the birth center. Furthermore, other healthcare professionals who might be needed to support the women during pregnancy and childbirth such as physiotherapists and psychiatrists are available when needed. The birth center will offer comprehensive services and facilities during pregnancy and after birth to fulfill the individual wishes of different women. As a result of close collaboration with the hospital, anesthetic care is available. In case of medical complications, e.g., when the baby is premature or when a caesarian section is needed, the women will be transported to the hospital.

It is expected that women have high preferences for these features of the birth center as they make it possible to meet different needs (e.g., the possibility for the partner to stay overnight or an intimate atmosphere). The center will be unique as it will be established in the Medical Center Haaglanden, the clinical training hospital of The Hague, which ensures that women can immediately be transported to the hospital in case of medical complications. To reach its goals the birth center has three main objectives: (1) connecting the given care to the specific needs of different social groups; (2) providing prenatal and birth care, offering customized information about pregnancy and child birth to each social group and, ultimately, (3) countering the high perinatal mortality rates [2].

### Aim of this study

The aim of this study was to analyze the preferences of pregnant women living in the city of The Hague for services and facilities that could be offered in the new proposed birth center. Meeting the preferences of women in disadvantaged situations requires clarity about the factors that cause differences between ethnic groups. When such differences are found, it can confirm the need for specific birth care that fulfills personal wishes. Specifically, this study analyzed the influence of ethnicity in relation with education on birth center care preferences.

In this paper the three steps and objectives of this study were as follows:

1. To provide information about preferences among pregnant women for birth center care as proposed by the project;
2. To explore ethnic differences in the preferences for this specific form of birth center care, and finally;
3. To explore the mutual influence of ethnicity and education in the preferences for this specific form of birth center care.

## Methods

### Data collection

The data used in the study were based on a survey among pregnant women in The Hague (the Netherlands) that was conducted as part of the birth center project^5^. The data were collected in August 2011, before the realization of the birth center; however, details of the planned birth center were sufficiently available to present to the respondents and to examine their preferences for characteristics of such a center.

The questionnaire was developed in close collaboration with two midwives who were involved in the establishment of the birth center. These midwives aimed to gain more insights in the needs of their clients. The questionnaire consisted of two main modules. The first set of questions encompassed the social and cultural background of the participants. The second set of questions focused on the preferences for the services and facilities that would be provided in this birth center. Three subsets of items, totaling 24 different items, were presented to the respondents:

- five questions on preferences about receiving practical information during pregnancy;
- ten questions on different aspects of receiving comprehensive care during pregnancy and childbirth and;
- nine questions on preferences about the proposed accommodation in the birth center.

The approached population consisted of women living in a disadvantaged district on the outskirts of The Hague who had no experience with a birth center as yet. All women recruited for this study had either a desire to become pregnant or were pregnant and visited a midwifery practice for their regular check-up. Both women with and without children were included. Approaching women after this check-up was the most practical way to include as many women as possible. Almost all women visited their midwife for information about their pregnancy and the different possibilities involved in giving birth. The birth care professionals at the midwifery practice informed the women about the study and requested them to participate. This guaranteed the privacy of the women because they could complete the questionnaire on the spot and return it anonymously. Women who did not speak sufficient Dutch were assisted by a telephone interpreter. As a result, a substantial number of non-Dutch respondents participated, which was important to conduct the intended analysis and group comparisons for this study.

### Response and missing values

During 1 month, all respondents (*N* = 208) visiting the midwifery practice were approached by their own midwife. In total, 200 (96%) completed the questionnaire. All dependent variables had their own, sometimes large, proportion of missing values. To prevent the loss of too many respondents, first the missing values of the independent variables were removed in case of nominal variables, (having a partner), or replaced by the mean value in case of interval variables (age, number of children and health). This resulted in a total of 186 remaining respondents. Second, the missing values on the dependent variables were removed and the analyses were performed for each dependent variable separately. This resulted in a different number of respondents for each analysis, ranging between 168 and 175.

### Measurement

#### Preferences

To capture the respondents’ preferences and their dimensions as the dependent variables of this study, principal factor analysis (PFA) was applied on the 24 items of the questionnaire. KMO and Bartlett’s test indicated that it was possible to apply PFA (not shown in this article). Respondents rated the items on a scale ranging from 1 ‘*completely disagree*’ to 5 ‘*completely agree*’.

According to the PFA (on the basis of eigenvalues, the Kaiser criterion, scree test and the interpretation) three aspects could be constructed with 17 statements (Table 1 in Appendix). To create the latent aspects, the mean scores of the items were summed up to Likert scale items. The first latent aspect was labeled as ‘*practical information*’ and consisted of five statements; the second, ‘*comprehensive care*’ , consisted of six statements, and the third latent aspect, labeled as ‘*accommodation*’, was also based on six statements. The internal (scale) validity of the three dimensions was supported by Cronbach’s alphas of .77, .80 and .70.

#### Ethnicity and education

Ethnicity was based on a self-identification question: ‘*What is your ethnicity?’* The answer categories were ‘Dutch’ , ‘Turkish’ , ‘Moroccan’ , ‘Surinamese’ , and ‘Other’. Because of the low prevalence of Surinamese women in our study population (despite the fact that the share of Surinamese women in The Hague is high), this group was merged with the category ‘Other’.

Education was measured using the following question: *‘What is your highest level of education completed (with diploma or certificate)?*’ This variable was included in the analyses as the nominal variable as it was not normally distributed. The answer categories were divided into ‘Low’ i.e., no education, primary school or vocational training (in Dutch: VMBO/MAVO); ‘Medium’ , i.e., senior general secondary education (in Dutch: HAVO), pre-university education (in Dutch: VWO) and secondary vocational education (in Dutch: MBO); and ‘High’, i.e., higher vocational education (in Dutch: HBO) and university (in Dutch: WO).

#### Control variables

In the models of the multiple linear regression analysis, four control variables were used. The first control variable, age, was measured using the open-ended question, *‘What is your age?’* The number of children was also measured with an open-ended question: *‘How many children do you have?’* Both these variables were used in the analysis as interval variables. The presence of a partner was a third control variable. Respondents were asked to describe their family situation by choosing from four answer categories. We merged the categories together to create a dummy variable with ‘partner’ (‘living together’ and ‘married’) as the first category and ‘no partner’ (‘single’ and ‘divorced’) as the second category. Finally, the subjective health of the respondents was measured using the following question: *‘In general, how would you assess your health?’* The answer categories were ‘Bad’ , ‘Moderate’ , ‘Good’ , ‘Very good’ and ‘Excellent’. This variable was normally distributed and, therefore, included in the analysis as an interval variable.

#### Data analysis

Two analyses were conducted to examine the preferences for the proposed birth center care in The Hague and its expected relation with the ethnic background and educational level of the pregnant women who participated in the survey. Descriptive analyses were used to meet the first aim: to investigate the average preferences for the proposed birth center care of women in The Hague. The second aim was to study differences on the basis of ethnicity. Multiple linear regression analysis was applied to explore these possible differences. This kind of analysis avoids spurious effects and estimates the influence of ethnicity controlled for other predictors (i.e., age, having a partner, number of children and subjective general health). This results in a more rigorous test. In the regression models, attention was also given to the interaction between the background characteristics of ethnicity and education (the third aim). All analyses were performed using PASW Statistics 18.

## Results

### Descriptive analyses

Table 2 in (Appendix) presents the descriptive values for the dependent and independent variables. It shows that 35% of the respondents identified themselves as Dutch, 20% as Turkish and 18% as Moroccan. The remaining respondents had another Western or non-Western ethnicity (27%). Among the Dutch women, almost half had a higher education level (48%, Table 3 in Appendix). For the non-Dutch women, the results show that most Turkish women had low levels of education (42%, Table 3 in Appendix) and that most Moroccan women had a medium education level (46%, Table 3 in Appendix). Among the women with another Western or non-Western ethnicity, the number of women with either low or high levels of education was the same (both 35%, Table 3 in Appendix).

The following results show the preference scores of the pregnant women for the proposed birth center care in their city. The survey participants attached a relatively high value to receiving comprehensive care during pregnancy, childbirth and the postpartum period (*M* = 3.93, Table 2 in Appendix) and to non-clinical accommodations in the center (*M* = 3.87, Table 2 in Appendix). The majority of women also attached value to receiving practical information (*M* = 3.69, Table 2 in Appendix), but this seemed to be of less importance than the other two aspects.

Tables 4 to 7 in Appendix present the descriptive values of all control variables by ethnicity.

### Multivariate analyses

After the descriptive analyses, multiple linear regression was applied to examine the relation between the birth center preferences and ethnicity. We performed regression analyses for each of the three dimensions we distinguished in measuring the preferences for birth center care as presented before. After excluding the missing values on the independent variables, as described previously (page 6), a total of 168 respondents remained in the analysis.

Inspection of the explained variance by the R^2^ of the three regression models showed that this goodness-of-fit measure (the extent to which observed outcomes are replicated by the regression model) was generally low. For the dependent variable ‘*accommodation*’ , the R^2^ is between 10 and 15%; the explained variance of the other preference dimensions, ‘*practical information*’ and ‘*comprehensive care*’ , were below 7%.

**Fig. 1:**
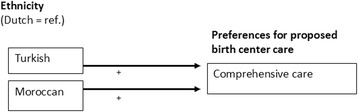
Effects of ethnicity on preferences for birth center care

Examining ethnic differences in preferences for the proposed birth center care based on multiple regression analysis (Fig. 1 and Table 8 in Appendix), we found that Turkish (B = 0.274) and Moroccan women (B = 0.346) attached more value to the importance of comprehensive birth center care during pregnancy, childbirth and the postpartum period. The relation between ethnicity on the other preference dimensions, ‘*practical information*’ and ‘*accommodation*’ , was not significant.

### Interaction between the background characteristics of ethnicity and education

**Fig. 2:**
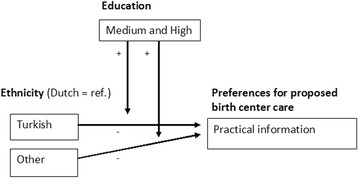
Interaction effect of ethnicity (Turkish and other) and education on preferences for the proposed birth center care

In the next step, we extended the multivariate analyses by estimating regression models in which the interaction term between education and ethnicity was included. The results of these analyses are presented in Table 9 in Appendix. The results showed four significant interactions between education and ethnicity on the preferences for the proposed birth center care. With regard to practical information, it seemed that Turkish women do not have stronger preferences than Dutch women (B = -0.400, not significant). However, when interpreting the interaction between ethnicity and education, it seemed that among the higher educated, Turkish women had stronger preferences than Dutch women (B = 0.735) (Fig. 2).

**Fig. 3:**
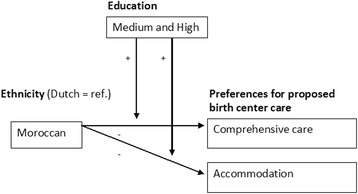
Interaction effect of ethnicity (Moroccan) and education on preferences for the proposed birth center care

For the women with another ethnicity, the results showed that they had less preferences for practical information than Dutch women (B = -0.517). Here, an interesting effect can be observed: among the higher educated, the gap in preferences between ‘other’ women and Dutch women was smaller and the sign of the effect had switched from negative to positive (B = 0.741). In other words, among the higher educated, women with another ethnicity had stronger preferences than Dutch women (Fig. 2). When considering the interaction between ethnicity and education on the aspects of preference for comprehensive care and accommodation, we found two significant effects. Moroccan women, in general, did not have stronger preferences for the comprehensiveness of the care or the accommodation than Dutch women (respectively B = -0.124 and B = -0.259, both not significant). However, among the higher educated, it seemed that Moroccan women had stronger preferences for these two factors than Dutch women (Fig. 3).

## Discussion

In this paper we investigated the preferences for a specific form of birth center care among pregnant women with different ethnic backgrounds living in The Hague, the Netherlands. The analyses were based on a survey conducted among 200 respondents, measuring preferences for three dimensions or aspects that are distinctive for birth care in the new proposed birth center: practical information, comprehensive care and comfortable accommodation. These dimensions were expected to match with the preferences and needs of Dutch and non-Dutch women. Non-Dutch or ethnic minority women were assumed to have stronger preferences for care offered in this birth center as they have specific needs and personal circumstances requiring customized and tailored services.

Overall, the women who participated in this study had relatively strong preferences for the proposed birth center care. These findings need to be interpreted with caution as it is difficult to categorize the results as positive or negative due to a lack of benchmark or norm. Our results seemed to indicate that, similar to women from other countries, women from the Netherlands were highly interested in the services that could be offered by a birth center [9–11]. However, Borquez and Wiegers’ (2006) study [12] showed that women in the Netherlands experienced home birth more positively than giving birth in a birth center; they perceived less pain, desired less pain-relieving medication, believed they knew their midwife better and rated their birth setting as ‘higher’. It is possible that, although women’s preferences are better met in a birth center, the actual experience of giving birth is better in their own trusted environment.

When focusing on the differences between ethnic groups, it showed that Non-Dutch women significantly judged the care in the proposed birth center to be better than Dutch women; this is because they had stronger preferences for the offered care during pregnancy, childbirth and the postpartum period. This can be explained by their different cultural customs and, as a result, their unfamiliarity with the Dutch healthcare system and the available home birth option. Besides, as some non-Dutch women do not speak sufficient Dutch, the offered maternity care does not always meet the health needs of these women and makes them in need of more guidance. The results of our study supports the expectation that for these specific groups, birth centers may provide personalized care, flexible options and the possibility to be involved in planning their own care [6–8]. As the survey data used in this study specifically focused on *preferences* for birth center care, it remains to be further researched whether or not the individualized care delivered in a birth center, in a culturally diverse urban area such as The Hague, will actually lead to improved birth outcomes. A study from the United Kingdom showed that care provided in a birth center positively influences pregnancy and child birth, e.g., women were less likely to have a C-section and more likely to carry to term [24].

A second result from our analysis was that, among the higher educated, non-Dutch women displayed a significantly stronger preference for all three dimensions of birth center care, i.e., extensive practical information, comprehensive care and comfortable accommodation. Education stimulates critical thinking and increases the health literacy skills of individuals. While bearing this in mind, it can be assumed that higher educated women take more initiative in searching or asking for information and are more demanding. As result, higher educated women are less satisfied with the offered birth care and/or have higher preferences for birth care because they tend to be more critical [25, 26]. This will trigger maternity care professionals to offer care in a dedicated and customized way for specific clients. In any case, it remains essential to educate non-Dutch women about pregnancy and childbirth to increase their involvement and to make them aware of the different settings in which they can give birth to their child.

It is important to bear in mind that this study was performed before the actual realization of the birth center in The Hague. The preferences of women were measured and analyzed for an as yet non-existing, proposed birth center in their city. Therefore, future (longitudinal) research should take a closer look at the advantages of birth centers and should examine to what extent birth centers actually fulfill the personal wishes of specific social groups such as ethnic minorities. When more insight into the contribution of different birth centers is available, evidence from policy evaluation research can lead to helpful suggestions concerning the design of birth centers in urban areas such as The Hague where inhabitants in disadvantaged situations are included.

Future research should also pay attention to factors other than ethnicity that probably play an important role in explaining women’s preferences for different aspects of birth center care. As our regression models showed, ethnicity and education were important determinants for preferences for birth center care. The low explained variance of the regression models also indicated, however, that many other explanatory factors are probably missing, e.g., household composition, income level, previous cultural and birth care experiences or the support received from family.

### Strengths and limitations

A major strength of this study was the high response number, also by non-Dutch women. Of the 208 pregnant women that were invited for the survey, 200 women (96%) completed the questionnaire, which means an almost perfect response rate. This was possibly due to the role of the midwives in asking the women to participate in the study. Another strength was that, through the support of the telephone interpreter and the personal way in which respondents were approached, many non-Dutch women (66%, *N* = 186), who often are reluctant to participate in similar studies, could be included as well. The interpreter enabled non-Dutch women to participate and to fill in the questionnaire completely. This provided unique information, often not available in survey research on this scale, also allowing comparison of women from dissimilar cultural backgrounds.

This study also has limitations. Due to the fact that mainly women in a disadvantaged situation participated, the findings might not be easily generalized to other projects as it is not representative for the Dutch population. Still, we were able to analyze substantial variations in our sample of women, which provide useful information for our research aim. As a result, a recommendation for future research is to include more municipalities with different social compositions.

Second, this study only focused on preferences of women for different characteristics of maternity care that were presented as being provided in the proposed birth center. Knowledge about the current satisfaction levels of pregnant women in The Hague with birth care elements is lacking. Women could, for instance, be dissatisfied with the comprehensiveness of care because they want to receive care in another manner. As all interviewed women visited the same midwifery practice, it is possible that their preferences partly reflect the care they are currently receiving. Moreover, other aspects that are important to (non-)Dutch women remain unknown. Therefore, future studies should conduct qualitative research among focus groups or individuals from multiple practices to gain insight into other important aspects.

Finally, the questionnaires that were filled in with the help of an interpreter could have led to social desirability in the answers, thereby introducing bias. Women could have felt obligated to please the telephone interpreter. However, social desirability during a telephone interview is lower compared to social desirability during a face-to-face interview.

## Conclusions

This study offered some important insights in the preferences for different dimensions of birth center care among pregnant women with different ethnic backgrounds. Overall, the importance of specific birth care that meets the personal wishes of vulnerable groups, emphasized in current policies in the Netherlands [2]^6^, was supported by this study. This also means that the actual need for birth centers differs between social groups. Especially for non-Dutch women, the realization of more birth centers with comprehensive services and facilities appears promising to better match their needs and enhance birth care quality. In general, is it recommendable that non-Dutch women should be more educated about maternity care provision in the Netherlands in order to increase their involvement during their pregnancy and birth and to ensure that they make clear their needs and wishes.

## Appendix

**Table 1:** The items ‘Practical information’, ‘Comprehensive care’, ‘Accommodation’ (created by using principal factor analysis (oblique rotation))

*Aspect*	*Item*	*Cronbach’s alfa*
Practical information	I want to receive practical information about getting pregnant in a healthy way	0.770
	I want to receive general information about the pregnancy	
	I want to receive practical information about childbirth	
	There must be a possibility to register my birth wishes	
	Information about what to expect during and after birth is very important	
Comprehensive care	The continuous assistance of a midwife from the moment of contractions is very important	0.802
	The continuous presence of midwives and obstetricians during labor and birth makes me feel comfortable	
	During labor and birth, it is reassuring that the hospital is nearby	
	It is important that I can stay a few days in the hospital or birth center during the postpartum period	
	The continuous presence of midwives and obstetricians during the postpartum period make me feel comfortable	
	In an emergency case, during the postpartum period, it is reassuring to have the hospital nearby	
Accommodation	A home-like atmosphere is important	0.708
	There must be colors on the walls	
	The presence of a waiting room is important	
	Flowers or plants should be present in the center	
	I want the possibility to invite my family for maternity visits	
	It is important that my partner can sleep in the same room before and during my labor

**Table 2:** Descriptive statistics preferences for proposed birth center care and ethnicity

	Frequency	Min.	Max.	Mean	S.D.	N
Ethnicity						
Dutch	64 (35%)					186
Turkish	38 (20%)					186
Moroccan	33 (18%)					186
Other ethnicity (includes respondents from non-Western and Western countries)	51 (27%)					186
Preferences for proposed birth center care						
*Value attached to:*						
Practical information		1	5	3.69	0.700	168
Comprehensive care		1	5	3.93	0.711	175
Accommodation		1	5	3.87	0.807	175

**Table 3:** The distribution of ethnicity and education

Education				
Ethnicity	Low	Medium	High	Total
Dutch	9 (14%)	24 (38%)	31 (48%)	64
Turkish	16 (42%)	13 (34%)	9 (24%)	38
Moroccan	8 (24%)	15 (46%)	10 (30%)	33
Other	18 (35%)	15 (29%)	18 (35%)	51
Total	51 (27%)	67 (36%)	68 (37%)	186

**Table 4:** The distribution of ethnicity and age

Age					
Ethnicity	25 and younger	26 to 30	31 to 35	36 and older	Total
Dutch	11 (17%)	24 (38%)	17 (27%)	12 (18%)	64
Turkish	6 (16%)	22 (58%)	7 (18%)	3 (8%)	38
Moroccan	15 (46%)	9 (27%)	7 (21%)	2 (6%)	33
Other	10 (20%)	14 (28%)	18 (35%)	9 (18%)	51
Total	42 (23%)	69 (37%)	49 (26%)	26 (14%)	186

**Table 5:** The distribution of ethnicity and partner

Partner			
Ethnicity	Partner	No partner	Total
Dutch	60 (94%)	4 (6%)	64
Turkish	36 (95%)	2 (5%)	38
Moroccan	32 (94%)	2 (6%)	33
Other	36 (71%)	15 (29%)	51
Total	163 (88%)	23 (12%)	186

**Table 6:** The distribution of ethnicity and number of children

Number of children					
Ethnicity	0	1	2	3 or more	Total
Dutch	12 (19%)	32 (50%)	14 (22%)	6 (9%)	64
Turkish	5 (13%)	17 (45%)	12 (32%)	4 (10%)	38
Moroccan	8 (24%)	12 (36%)	9 (28%)	4 (12%)	33
Other	13 (26%)	27 (44%)	10 (20%)	5 (10%)	51
Total	38 (20%)	84 (45%)	45 (25%)	19 (10%)	186

**Table 7:** The distribution of ethnicity and health

Health				
Ethnicity	Moderate to bad	Good to very good	Excellent	Total
Dutch	1 (2%)	57 (89%)	6 (9%)	64
Turkish	9 (24%)	23 (60%)	6 (16%)	38
Moroccan	3 (9%)	28 (85%)	2 (6%)	33
Other	6 (12%)	42 (82%)	3 (6%)	51
Total	19 (10%)	150 (81%)	17 (9%)	186

**Table 8:** Linear regression analyses of preferences for birth center care

	Practical information	Comprehensive care	Accommodation
	B	B	B
Ethnicity (ref. = Dutch)			
Turkish	0.131	0.274*	0.041
Moroccan	0.191	0.346*	0.221
Other	0.015	0.097	-.091
Education (ref. = low)			
Medium	0.205	0.317*	0.336*
High	0.102	0.220	0.296*
Age	0.002	−0.004	−0.011
Partner (ref. = no partner)	−0.036	0.069	0.241
Number of children	−0.068	0.010	−0.014
Health	−0.026	−0.011	0.099
Intercept	3.659***	3.665***	3.432***
R^2^	0.035	0.068	0.101
N^a^	168	175	175

**p <* 0.05; ***p <* 0.01; ****p <* 0.001

^a^After removing the missing values of the independent variables, there were 186 respondents eligible for analysis. The number of missing values on each of the dependent variables varied, which is why the analysis was performed for each dependent variable separately. This resulted in a different number of respondents for each analysis

**Table 9:** Linear regression analysis of preferences for birth center care (practical information, comprehensive care and accommodation)

	Practical information		Comprehensive care		Accommodation	
	Model A	Model B	Model A	Model B	Model A	Model B
	B	B	B	B	B	B
Ethnicity (ref. = Dutch)						
Turkish	0.131	−0.400	0.274*	0.003	0.041	−0.334
Moroccan	0.191	0.223	0.346*	−0.124	0.221	−0.259
Other	0.015	−0.517*	0.097	−0.185	-.091	−0.151
Education (ref. = low)						
Medium	0.205	−0.097	0.317*	0.184	0.336*	0.234
High	0.102	−0.412	0.220	−0.221	0.296*	−0.065
Age	0.002	0.001	−0.004	−0.001	−0.011	−0.009
Partner (ref. = no partner)	−0.036	−0.060	0.069	0.071	0.241	0.257
Number of children	−0.068	−0.085	0.010	0.010	−0.014	−0.006
Health	−0.026	−0.037	−0.011	0.004	0.099	0.090
Turkish*Medium		0.614		0.120		0.436
Turkish*High		0.735*		0.470		0.542
Moroccan*Medium		−0.452		0.214		0.340
Moroccan*High		0.369		1.049**		0.898*
Other*Medium		0.587		0.091		−0.379
Other*High		0.741*		0.502		0.276
Intercept	3.659***	4.142***	3.665***	3.793***	3.432***	3.591***
R^2^	0.035	0.113	0.068	0.112	0.101	0.146
N^a^	168	168	175	175	175	175

**p* < 0.05; ***p* < 0.01; ****p* < 0.00

^a^After removing the missing values of the independent variables, there were 186 respondents eligible for analysis. The number of missing values on each of the dependent variables varied, which is why the analysis was performed for each dependent variable separately. This resulted in a different number of respondents for each analysis

## Footnotes

1 The percentage of home births in the Netherlands is decreasing. A possible cause for this trend is a slowly increasing number of medium risk situations due to an increase in local protocols for risk perceptions. Women with a medium risk indication can no longer choose where they want to give birth. As a consequence, there is an increase of births in obstetric hospital units.

2 The Steering Committee on Pregnancy and Childbirth was established as an answer to the results of the first European Perinatal Health Report (2008) [27], in which the relatively high perinatal mortality rates of the Netherlands were shown. Its aim was to increase the quality of the maternity care together with various other implemented policy measures.

3 In the Netherlands, almost all women who give birth, whether at home or in hospital, receive care from a maternity care assistant (MCA) at home, between 3 and 8 h a day, up to eight consecutive days following the birth. When women give birth at home, the MCA is also expected to be present at the time of birth, to give assistance to the midwife or GP and to take care of the mother and her child for at least 2 h after the placenta has been born. When women give birth in a hospital, the MCA is present when mother and newborn return home from the hospital. The care that is provided by maternity care assistants is part of the standard insurance package [28].

4 In the Netherlands, maternity care is organized in three levels; primary, secondary and tertiary care. The level of care is organized according to the presence or absence of risk factors in medical and/or obstetrical history. The primary care is formed by midwives and GPs (with a focus on low-risk women); the secondary care is formed by obstetricians and specialized ‘clinical’ midwives in general hospitals (for women with an a priori high risk profile); and the tertiary care consists of obstetricians in academic hospitals. In this article we focus on the collaboration between primary and secondary birth care.

5 From autumn 2009 until April 2014, the Dutch Ministry of Health, Welfare and Sports commissioned the ‘Primary focus’ program, which was organized by the Dutch Organization for Health Research and Development. The program was aimed at improving integrated care and enhancing our knowledge of collaboration efforts in primary health care [29]. As part of the program, projects concerning birth care in Dutch birth centers were monitored with an emphasis on interprofessional collaboration. In the survey used for this paper, questions on preferences in child birth care were added.

6 The current policies refer to the national policy of the Netherlands that is focused on customized care.
